# Tumor budding as a predictor for prognosis and therapeutic response in gastric cancer: A mini review

**DOI:** 10.3389/fonc.2022.1003959

**Published:** 2023-01-23

**Authors:** Chi Xue, Yuwei Du, Yuegang Li, Huimian Xu, Zhi Zhu

**Affiliations:** Department of Surgical Oncology, First Affiliated Hospital of China Medical University, Shenyang, China

**Keywords:** EMT, gastric cancer, prognosis, tumor budding, therapeutic response

## Abstract

In recent years, the role of tumor budding in gastric cancer has received increased attention across a number of disciplines. Several studies have found associations between tumor budding and the prediction of lymph node metastasis in early gastric cancer, prognosis of advanced gastric cancer, predictors of therapeutic response to immune checkpoint inhibitors, such as microsatellite instability (MSI), and therapeutic targets of molecular targeted therapy, such as human epidermal growth factor receptor 2 (HER-2). Therefore, tumor budding is a major element in the formulation of risk stratification and precision medicine strategies for patients with gastric cancer.

## 1 Introduction

According to the 2020 WHO statistics (1), gastric cancer ranks fifth in incidence and fourth in mortality globally among all cancers. The choice of treatment modality and prognostic criteria for gastric cancer often depends on the TNM staging system. In recent years, tumor budding, a general clinicopathological feature of tumor aggressiveness, invasion, and poor prognosis, has attracted increasing attention. This pathologic phenomenon has also been observed in other tumors, such as extrahepatic cholangiocarcinoma (ECA) (2), pancreatic ductal carcinoma (PDC) (3), oral cancer (4), and cervical squamous cancer (ECSC) (5); thus, tumor budding is not unique to gastric cancer but is widespread among all tumors.

This review will address the concept of tumor budding, the molecular mechanism underlying this pathologic phenomenon in gastric cancer, and its role in predicting the prognosis and therapeutics of gastric cancer, to provide a new modality and reference for the individualized diagnosis and treatment of patients with gastric cancer.

## 2 Overview

**Figure 1:**
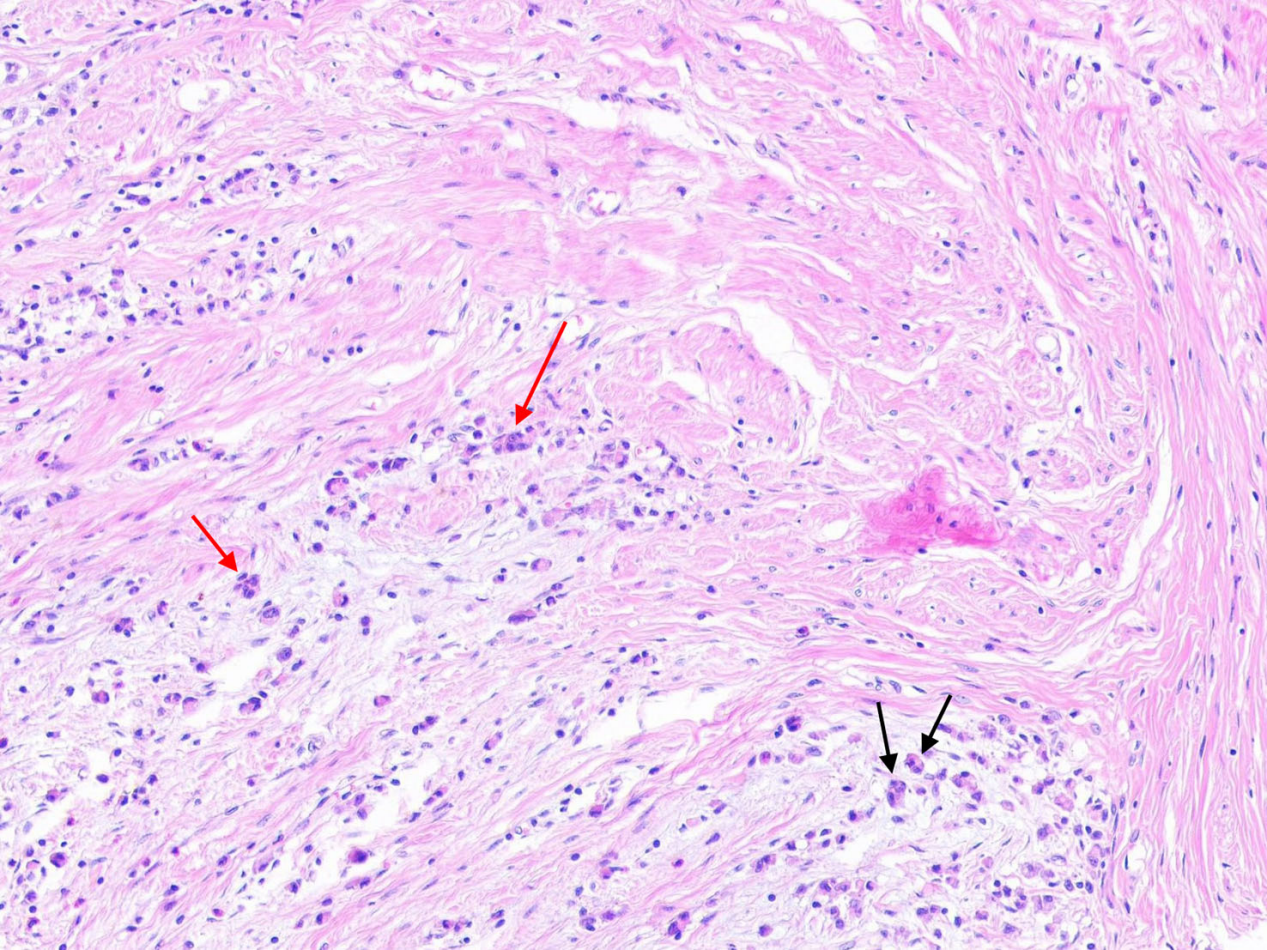
The black arrow represents tumor budding in gastric cancer (single tumor cells or clusters of up to four tumor cells), and the red arrow represents poorly differentiated cluster (PDC) in gastric cancer (five or more cells).

According to the recommendations for reporting tumor budding in colorectal cancer based on the International Tumor Budding Consensus Conference (ITBCC) 2016 (6), tumor budding is defined as a single cell or clusters of up to four cells at the cancer invasion margin and can be stratified into peritumoral budding [(PTB), tumor budding at the tumor front] and intra-tumoral budding [(ITB), tumor budding in the tumor center and surrounded by tumor stroma (7)] (i.e., “**Figure 1**”). PTB can only be assessed using surgical resection specimens, whereas ITB can be assessed using biopsies and resection specimens.

Although the ITBCC provides a definition of the cell number and location of tumor budding, it does not specify the pathologic changes that accompany tumor budding. Thus, there has been minimal consensus about the histopathological changes related to tumor budding. Gabbert et al. (8) reported the pathologic changes of tumor budding visualized by light microscopy as early as 1985; at the invasion front, the regular architecture of differentiated carcinomas is lost. Here, the tumor glands are separated from each other and are composed of flat-to-cuboidal tumor cells. At the foremost border of the invasion front, there are no tumor glands, but there are isolated tumor cells. Some of these tumor cells undergo mitosis and aggregate into very small tumor cell complexes. The cell shape the isolated tumor cells at the foremost invasion front is extremely variable and ranges from round or oval to sandglass-like or arrow-like. Unlike the study of Gabbert et al., in 2014, Bronsert et al. (9) found that many tumor buddings were interconnected and ultimately connected to the main tumor branches in pancreatic ductal adenocarcinoma, colorectal adenocarcinoma, liver metastasis of colorectal adenocarcinoma, lung adenocarcinoma and invasive ductal breast cancer, when reconstructed in 3D. In 2020, Yoshizawa et al. (2) confirmed that high-grade tumor budding had more branch points than low-grade tumor budding (median, 26 vs. 20, p = 0.021) and longer mean protrusion length (median, 53.3 vs 32.1 μm, p < 0.001). Some scholars (10) have also proposed that tumor nodules within 1 mm of the tumor edge should not be regarded as tumor budding, because tumor nodules may be linked to tumor tissue in deeper sections and should be regarded as “discontinuous diffusion” of tumor tissues.

Currently, the ITBCC group (6) recommends the use of the following three-tier system, as used by the Japanese Society for Cancer of the Colon and Rectum: low-grade budding (Bd 1), 0-4 buds; intermediate grade budding (Bd 2), 5-9 buds; and high grade budding (Bd 3), 10 or more buds. However, in practical applications, different tumor have different grading criteria for tumor budding. For example, tumor budding is divided into two grades in head and neck, oral, and cervical cancers (11–13) [low grade of budding (0-5 buds) and high grade of budding (more than 5 buds)]. In upper urothelial carcinoma and pancreatic ductal adenocarcinoma (14, 15), less than 10 buds is considered low grade of budding, while more than 10 buds is considered high grade. In colon cancer, Zlobec et al. (16) found that there was a significant difference when BD0(0 buds) was compared statistically to BD1 (1–4 buds) for pT, TNM, tumor grade, and lymphatic, venous, and perineural invasion (p < 0.01, all). Because of these findings, they recommend that BD0 should be considered for inclusion in future ITBCC guidelines. In muscle invasive urothelial carcinomas, Lorenzo Soriano et al. (17) and Seker et al. (18) determined the critical value of tumor budding through receiver operating characteristics (ROC) curve, which also provided a new idea for the formulation of grading standard for tumor budding in gastric cancer in the future. Furthermore, there is little agreement on whether the type of tumor budding should be evaluated as PTB or ITB. Some scholars believe that for certain tumors, such as PDC, the scope of the tumor invasion edge cannot be clearly defined because of the small extent of resection. Thus, ITB should be used to evaluate the amount of tumor budding.

In gastric cancer, the grading system recommended by ITBCC has been used in many studies. However, due to the inability to distinguish the pathologic differences between diffuse-type gastric cancer and tumor budding, many studies cannot apply the tumor budding classification system to diffuse-type gastric cancer. Therefore, many studies are limited to intestinal-type gastric cancer, which can be distinguished from tumor budding (19). Although the number of patients with intestinal-type gastric cancer account for more than 50% of the total number of those with gastric cancers, the prognosis of patients with diffuse-type gastric cancer is worse than those with intestinal-type gastric cancer (20), which introduces bias in studies of the relationship between tumor budding and patient prognosis. Therefore, it is necessary to establish standard grading system for tumor budding in the future research of gastric cancer

## 3 Molecular mechanism underlying the pathogenesis of tumor budding in gastric cancer

### 3.1 Epithelial-mesenchymal transformation is the initiating process of tumor budding

Sun et al. (21) reported that ZBTB7A is highly expressed at the edge of enteric-type gastric cancers. ZBTB7A acts as a transcription factor that inhibits the expression of the Arf tumor suppressor gene, which results in decreased P53 activity. At the same time, high Arf expression highly correlates with tumor budding, but negatively correlates with E-cadherin expression. The loss of E-cadherin expression will be manifested by tumor cells dissociating from each other, as they lose cell-cell adhesive junctions and acquire mesenchymal characteristics, which also contributes to the phenomenon of tumor budding (22). Furthermore, E-cadherin expression is also inhibited due to the high expression of the upstream TGF-β signaling pathway. Increased levels of TGF-β may also contribute to acquiring metastatic ability, as it enables gastric cancer cells to destroy and penetrate basement membrane barriers (23), enabling tumor cells to “escape” into the stroma and eventually form tumor buds. The change in E-cadherin expression is not simply quantitative, however, because although tumor cells lose E-cadherin membrane expression, there is a simultaneous increase in cytoplasmic expression of the protein. This allows to study EMT directly during tumor budding in tumor cell clusters of different cell numbers, demonstrating that the fraction of cells with cytoplasmic E-cadherin staining is significantly increased in smaller cell clusters, whereas the fraction of cells with mixed (cytoplasmic/membrane) and membrane expression patterns decreased with decreasing tumor cell cluster size (24).

### 3.2 Anoikis resistance promotes tumor bud survival

Cells express a variety of cell-surface adhesion molecules that mechanically act as contact points between cells and the extracellular matrix or adjacent cells and initiate intracellular signaling pathways that regulate important cellular events, including survival and proliferation. Normal cells undergo apoptosis in the absence of extracellular matrix attachment. This type of cell death is known as anoikis (25). Tanaka et al. (26) showed that the level of Trkb expression at the gastric cancer invasion front and in tumor budding cells was significantly higher than that in tumor cells in the gastric cancer center, with a significant positive correlation between the level of Trkb expression at the tumor invasion front and tumor budding (p = 0.0023). However, there was no significant correlation between tumor budding and Trkb expression in the gastric cancer center (p = 0.0997). Another study (27) showed that the BDAF/Trkb pathway inhibits the expression of E-cadherin in cells and promotes epithelial-mesenchymal transformation, the proliferative activity of tumor cells, and anoikis resistance. Thus, these results suggest that after tumor cells lose their attachment sites and attain mesenchymal cell characteristics through epithelial-mesenchymal transformation, the tumor cells can continue to survive in the mesenchyme and metastasize to distant places through the high expression of Trkb, eventually forming tumor buds.

### 3.3 Changes in the immune microenvironment inhibit tumor buds clearance

Zhang et al. (28) analyzed immune cell infiltration in the tumor budding microenvironment of gastric cancer. They observed a negative correlation between the density of tumor budding and tumor-infiltrating lymphocytes (TILs) in the budding area, tumor stroma, and parenchyma. The number of TILs around the tumor budding was reduced compared with TILs in the non-budding region (p < 0.001). Additionally, the number of TILs in turn changed from non-budding area CD8+>FOXP3+>OX40+> GrB + T cells to FOXP3+>CD8+>OX40 + T > GrB + T cells in budding area. CD8 surface antigen-expressing cytotoxic T lymphocytes are the most effective cells in the antitumor immune response. The abundance of CD8 + TILs positively correlates with better prognosis (HR = 0.77, 95% CI = 0.63-0.95) (29). Regulatory T cells (Tregs) are characterized by the expression of the transcription factor Foxp3, which is essential for the prevention of autoimmunity, maintenance of immune homeostasis, and regulation of immune responses to self and foreign antigens (30). Both tumor cells and Tregs can have high expression levels of TGF-β, which upregulates Foxp3 and Treg functional polarization in CD4+T cells and transforms macrophages from the M1-to-M2 type (31). Therefore, in the tumor budding area, tumor cells can increase TGF-β levels to increase Treg levels and the number of M2-type macrophages. This further reduces the immune response and the immune microenvironment conducive to M2-type macrophage growth, to benefit tumor cells for peripheral transfer and not be cleaned by immune cells.

**Figure 2:**
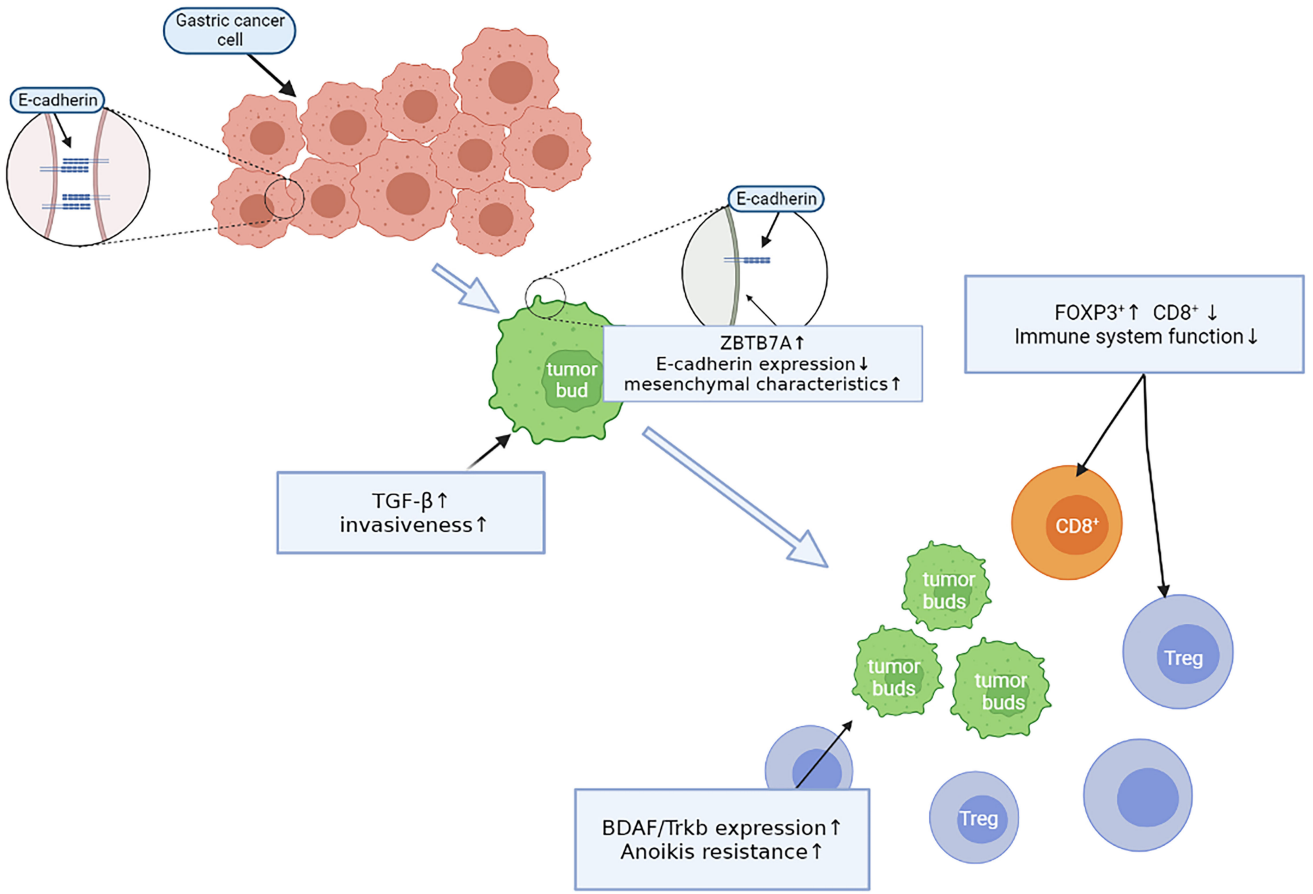
The continuous process of tumor budding.

These experiments revealed that during the early tumor budding process in gastric cancer, all steps are not isolated but are rather closely related and complementary through a complete and continuous process (**Figure 2**).

### 3.4 Prognosis and treatment of tumor budding in gastric cancer

#### 3.4.1 Prognosis of early gastric cancer

Based on the 2016 ITBCC study (6), tumor budding was identified as an independent predictor of lymph node metastasis in patients with pT1 colorectal cancer. Simultaneously, it was widely recognized by academics that the occurrence and development of tumor budding is significantly correlated with the highly invasive properties of tumor cells, and the degree of occurrence and development is highly correlated with lymph node metastasis in early gastric cancer. As early as 2000, Matsumoto et al. (32) indicated a significant relationship between irregular narrowing or tumor buddings in the third layer on EUS and submucosal tumor invasion(p < 0.01). The investigators recommended for lymph node removal to be considered, even when the endoscopy and biopsy show that the lesions present indications for therapeutic endoscopic treatment. In 2015, Gulluoglu et al. (33) conducted a study that involved 126 patients with early gastric cancer after radical total and subtotal gastrectomy. The clinicians reported that tumor budding was the only independent risk factor for lymph node metastasis in pT1a and pT1b gastric cancer. In 2019, Du et al. (34) also showed that tumor budding was a significant risk factor for lymph node metastasis in patients with early gastric cancer. Furthermore, in some early gastric cancers, lymph node metastasis was absent when there was no tumor budding (47 patients with submucosal early gastric cancer from the cardia, 15 with submucosal early stage gastric cancer <1.0 cm in size, and 17 cases of well-differentiated tubular/papillary early stage gastric cancer <1.0 cm in size). In 2021, Yim et al. (35) found that mTB (modified tumor budding, which excludes the signet ring cell component) was superior to traditional tumor budding (dAUC, 0.085 and 0.087 vs. 0.054 and 0.057) in predicting lymph node metastasis,which can significantly increase lymph node metastasis prediction accuracy in early gastric cancer.

Overall, these results indicate that tumor budding can be used as a predictor of lymph node metastasis in early gastric cancer and as a potential predictive factor to provide precise treatment strategies for patients with early gastric cancer (**Table 1**).

**Table 1:** Results of tumor budding in early gastric cancer.

Researcher	Year	Sample source	Pathologic stage	Number of samples	P value of lymph node metastasis	Conclusion
**Du**	2019	Surgical specimen	pT1b EGC	621	<0.01	Tumor budding is a significant high-risk factor for lymph node metastasis in submucosal early gastric carcinoma
**Yusuke**	2000	Endoscopically resected specimen	pT1a & pT1b EGC	75	\	The findings of irregular narrowing or tumor buddings in the third layer on EUS and submucosal invasion of the tumor has a significant relationship (P<0.01)
**Yim**	2021	Surgical specimen	pT1a & pT1b EGC	289	0.03	Tumor budding was the most predictive independent factor for lymph node metastasis, for groups containing all kinds of EGCs. Meanwhile, excluding signet ring cell from tumor budding significantly increased its lymph node metastasis prediction ability, compared to conventional tumor budding.
**Gulluoglu**	2015	Surgical specimen	pT1a & pT1b EGC	126	0.04 in pT1a	Presence of tumor budding was the only variable that remained statistically significant as an independent marker for node pT1a & pT1b positivity of EGC
					<0.0001 in pT1b	

#### 3.4.2 Prognosis of advanced gastric cancer

Nearly all of the studies on tumor budding in advanced gastric cancer have indicated that tumor budding is an important predictor of gastric cancer prognosis; however, each study had a different focus. Kucuk et al. (35) and Pun et al. (36) discovered that tumor budding was significantly related to pathologic stage and lymph node involvement (p < 0.01 & p < 0.001 and p < 0.05 & p = 0.004). In a study that involved 104 patients with surgically-resected gastric adenocarcinoma, Olsen et al. (36) reported that patients with high tumor budding were more likely to have nerve infiltration than patients with low budding (52% vs. 11%, p = 0.002), lower T stage (70% vs. 10%, p < 0.001), and higher recurrence rate (27% vs. 0, p = 0.007). In the study on the relationship between diffuse gastric cancer with a high budding grade and intestinal gastric cancer, while no significant differences in the number and recurrence rate of lymph node metastasis was observed, intestinal gastric cancer had higher lymphovascular infiltration than diffuse gastric cancer (76% vs. 39%, p = 0.002). Thus, gastric cancer with a higher tumor budding grade has a stronger invasion ability, worse prognosis, and higher recurrence probability. Dao et al. (37) found that patients with a lower budding grade (grade 1 and 2) had a longer overall survival time than those with a higher budding grade (grade 3 and above) [(OS), 78.07 ± 2.15 vs. 33.87 ± 3.48 months]. With respect to 5-year disease-free survival (DFS), patients with budding grades 1 and 2 showed DFS rates of 95.0% and 84.7%, respectively, while all patients with a higher budding grade died before 5-years, with a statistically significant difference between the low and high budding grade groups (p < 0.001). Based on the connection between tumor budding and clinicopathology, Dao et al. proposed that tumor budding could be used as a tool for risk stratification prediction, which would be of great significance for guiding tumor follow-up treatment.

With regard to pathological characteristics, Qi et al. (38) studied PTB and ITB of gastric adenocarcinoma specimens and discovered that ITB was present in more patient tumor tissues than PTB (92.8% vs. 33.3%). Compared with ITB alone, patients with PTB and ITB had lower overall survival (42.43 vs. 54.62 months, p = 0.033) and a worse prognosis (p < 0.001). Therefore, ITB has application value in pathological biopsies, prediction of lymph node metastasis, and prediction of preoperative staging. However, the prognostic difference between ITB and PTB has not been compared in detail; therefore, it remains necessary to further explore which type of tumor budding form should be used for pre- and postoperative prognosis and staging evaluations. Furthermore, Szalai et al. (39) compared the ability of tumor budding and poorly differentiated clusters to predict prognosis and lymph node metastasis. The analyses showed higher tumor budding has poorer overall survival and more lymph node metastasis in the total cohort (p = 0.014 & p = 0.038) and in intestinal-type adenocarcinomas (p = 0.005 & p = 0.019). In contrast to tumor budding, no significant association was found between poorly differentiated clusters and the occurrence of lymph node metastasis, tumor stage, or survival. The results of this study further reveal the superiority of tumor budding in predicting prognosis and lymph node metastasis in patients with gastric cancer.

In patients receiving adjuvant chemotherapy, Jesinghaus et al. (40) reported that tumor budding was associated with many clinicopathological characteristics after neoadjuvant chemotherapy (ypT (p < 0.001), ypN (p = 0.045), and ypM stage (p = 0.050)). In parallel, tumor budding can stratify the prognosis of patients after adjuvant chemotherapy. Notably, a Kaplan-Meier survival curve analysis revealed significant differences in survival between the three grades of tumor budding in patients after adjuvant chemotherapy (p < 0.001). Patients whose tumors were assigned to the Bd1 subgroup had a mean OS of 51.7 months (95% CI:46.5–56.8 months) compared to 37.4 months for Bd2 (95% CI:30.3–44.5 months; HR:3.48, 95% CI:1.57–7.73) and 28.1 months for Bd3 carcinomas (95% CI:23.2–33.1 months; HR:6.26, 95% CI:3.06–12.81).

Therefore, tumor budding in patients who are operable (with or without neoadjuvant chemotherapy) can effectively predict prognosis and lymph node metastasis and stratify patient prognosis. However, few studies have investigated the prognostic relationship between tumor budding and advanced unresectable gastric cancer, and further research is needed in this regard (**Table 2**).

**Table 2:** Results of tumor budding in advanced gastric cancer.

Researchers	Year	Sample source	Number of samples	Pathological classification	Budding type	Budding grade	P value of lymph node metastasis	OS (low grade VS high grade, month)	Conclusion
**Qi**	2020	Surgical specimen	170	Intestinal carcinomas	ITB	0-12 low grade ≧13high grade	<0.001	\	The presence of ITB predicts lymph node metastasis, which suggests that ITB is clinically relevant for predicting lymph node status
				Intestinal carcinomas	PTB	0-8 low grade ≧9 high grade	0.003	\	
**Olsen**	2017	Surgical specimen	52	Intestinal carcinomas	ITB & PTB	High grade tumor bud score ≧1 Low grade tumor bud score <1	<0.001	\	Tumor budding was significantly associated with tumor differentiation, lymphovascular space invasion, T-stage, and N-stage in a univariate analysis of the cohort
**Dao**	2020	Surgical specimen	109	Gastric adenocarcinoma	ITB & PTB	0-9 low grade >10 high grade	<0.001	78.91 ± 2.00 VS 36.69 ± 3.45	Tumor budding may provide a unique predictive hallmark to stratify risk categories of patients with gastric cancer
**Ulase**	2019	Surgical specimen	456	Gastric adenocarcinoma	ITB & PTB	0 tumor budding absent 1-9 low grade ≧10 high grade	<0.001	48.0 vs 12.7 vs 12.8	Tumor budding was associated with various adverse clinicopathological features and patient outcomes
**Kemi**	2019	Surgical specimen	583	Intestinal carcinomas	ITB & PTB	0-9 low grade ≧10 high grade	\	\	High tumor budding is an independent prognostic factor in gastric adenocarcinoma, more specifically, in intestinal-type gastric adenocarcinoma. Assessment of tumor budding in diffuse-type gastric adenocarcinoma is not recommended
**Jesinghaus**	2022	Patients received neoadjuvant chemotherapy prior to their resection	167	Intestinal carcinomas	ITB & PTB	0-4 low grade 5-9 intermediate grade ≧10 high grade	\	51.7 vs 37.4 vs 28.1	The assessment of tumor budding according to the ITBCC criteria provides valuable prognostic information in the post-neoadjuvant setting of intestinal-type gastric cancer and may be a considerable substitute for the conventional grading system in gastric cancers after neoadjuvant therapy
**Pun**	2022	Surgical specimen	76	Gastric adenocarcinoma	ITB & PTB	0-4 low grade 5-9 intermediate grade ≧10 high grade	0.005	\	Tumor budding has prognostic value in intestinal-type gastric adenocarcinoma
**Jesinghaus**	2021	Surgical specimen	43	Gastric adenocarcinoma	ITB & PTB	1-4 low grade 5-9 intermediate grade ≧10 high grade	\	\	Tumor budding was statistically significantly related to pathologic stage, lymph node involvement, and grade. The finding also suggest that tumor budding can be applied to gastric cancer and it might contribute to the standardisation of diagnosis and prognostic factors
**Szalai**	2022	Surgical specimen	290	Gastric adenocarcinoma	ITB & PTB	0 Bd 0 1-4 Bd 1 5-9 Bd 2 ≧10 Bd 3	<0.0001	\	Tumor budding is an independent prognostic factor for survival in gastric cancer, especially in intestinal-type adenocarcinomas. However, there was no significant association between PDC and the occurrence of lymph node metastasis, tumor stage, and survival

### 3.5 Therapeutic targets of tumor budding in gastric cancer

Ulase et al. (41) analyzed tumor budding in 456 surgically resected specimens and found that tumor budding grade significantly correlated with MSI and HER-2. At the same time, there was a significant association between tumor budding and MET status, but in contrast to HER-2 and MSI, gastric cancer with a high budding grade tended to be MET-positive more frequently than tumors with a low budding grade. Heckl et al. (42) studied the relationship between insulin receptors and gastric cancer and found that the expression of insulin receptors in gastric cancer cells negatively correlated with tumor budding (p < 0.001) and significantly correlated with HER-2 status (p = 0.002). Insulin receptor expression was found to be higher in HER-2+ tumor cells, which suggests that tumor budding not only predicts insulin receptor status, but also that the combination of HER-2 inhibitors and insulin receptor blockers (or metformin) may provide a potential treatment for patients with tumor budding at the corresponding grade in the future.

At present, there are few studies on the relationship between tumor budding, MSI, and PD-1 in gastric cancer, however, it has been widely described in colorectal cancer and other tumor types. A study in colon cancer by Jass et al. (43) found that the frequency of both somatic APC mutation and tumor budding increased pari passu in cancers stratified as sporadic MSI high (MSI-H), hereditary non-polyposis colorectal cancer (HNPCC), MSI low (MSI-L), and microsatellite stable (MSS). Notably, this finding explains why a lack of tumor budding correlates with improved prognosis in MSI-H colorectal cancer. However, while few studies have investigated the relationship between tumor budding and MSI and PD-1 in gastric cancer, the relationship has been widely described in colorectal cancer and other tumor types. In colorectal cancer, tumor budding was found to strongly correlate with PD-L1 positive MSI-H. The study of Korehisa et al. (44) reported that PD-L1 expression in tumor cells (PD-L1 (T))-positive MSI-H CRCs did not correlate with budding graded 2 or 3 (p = 0.34); however, PD-L1 expression in tumor-infiltrating myeloid cells in stroma (PD-L1 (I))-positive MSI-H colorectal cancers significantly correlated with budding grades 2 or grade 3 (p = 0.043). However, the investigation of Kim et al. (45) did not observe such a correlation. The researchers reported that PD-L1+(T) tumors in MSI-H colorectal cancers significantly correlated with tumor budding-positivity (p < 0.001). The differential findings may be due to the different grades of tumor budding that were investigated in the two studies. Therefore, it is necessary to further analyze the relationship between tumor budding, MSI, and PD-L1 expression according to the standard classification criteria for tumor budding, in both colorectal and gastric cancers.

Thus, the therapeutic targets associated with tumor budding explains how tumor budding appears as a proliferative phenotype and invasive phenomenon and provides a more perfect risk assessment and grading treatment strategy for patients with tumor budding. Thus, the treatment of patients with tumor budding follow-up may provide a possible direction. Meanwhile, although tumor budding has been correlated with immunotherapy targets such as PD-1/PD-L1 and MSI in other tumors, due to the particularity of gastric cancer histopathology, it remains necessary to prove the potential relationship between gastric cancer tumor sprouting and immunotherapy targets through further experiments. Such investigations may also help to resolve controversial issues in related fields.

## 4 Discussion

At present, tumor budding is an important prognosticator in gastric cancer, but further investigation is warranted to address outstanding questions. The classification system for tumor budding in gastric cancer is imperfect, and many studies have used the colon cancer staging system for prognosis assessment. Owing to the histologic classification of gastric cancer, this system cannot be applied to all patients with gastric cancer, which also presents certain limitations. The classification system for tumor budding is also of great significance for the precise treatment of patients with gastric cancer. At the same time, the evaluation of tumor specificity should choose the type of tumor budding (i.e., only evaluate ITB or PTB, simultaneously evaluate PTB and ITB, or exclude certain types of cells, such as signet ring cells), and the specific parameters still need further evaluation and formulation. Yim et al. provided a possible solution for early gastric cancer, however, this solution needs to be investigated for relevance in advanced gastric cancer. Third, there is a relative paucity of high-quality research into the molecular biological mechanisms underlying tumor budding in gastric cancer. Several unresolved questions also remain regarding the continuous pathologic development process of tumor budding. There is also a paucity of literature specifically relating to predisposing factors for tumor budding, which will contribute to our understanding of tumor budding.

With the growing recognition of tumor budding in gastric cancer and the development of related technologies, many questions related to this will be answered in the near future, which may promote and improve the continuous development of gastric cancer diagnosis and treatment.

## Author contributions

CX and YD drafted the manuscript; HX and ZZ polished the manuscript multiple times to significantly enhance the quality; YL, YD, and ZZ gave useful suggestions. All authors contributed to the article and approved the submitted version.
